# A Bioluminescent 3CL^Pro^ Activity Assay to Monitor SARS-CoV-2 Replication and Identify Inhibitors

**DOI:** 10.3390/v13091814

**Published:** 2021-09-12

**Authors:** Cyrille Mathieu, Franck Touret, Clémence Jacquemin, Yves L. Janin, Antoine Nougairède, Manon Brailly, Magalie Mazelier, Didier Décimo, Virginie Vasseur, Aymeric Hans, José-Carlos Valle-Casuso, Xavier de Lamballerie, Branka Horvat, Patrice André, Mustapha Si-Tahar, Vincent Lotteau, Pierre-Olivier Vidalain

**Affiliations:** 1 CIRI, Centre International de Recherche en Infectiologie, Team Immunobiology of the Viral Infections, Univ Lyon, Institut National de la Santé et de la Recherche Médicale (Inserm), U1111, Centre National de la Recherche Scientifique (CNRS), UMR5308, Ecole Normale Supérieure de Lyon, Université Claude Bernard Lyon 1, 69007 Lyon, France; cyrille.mathieu@inserm.fr (C.M.); brailly.manon@gmail.com (M.B.); magalie.mazelier@gmail.com (M.M.); didier.decimo@inserm.fr (D.D.); branka.horvat@inserm.fr (B.H.); 2 Unité des Virus Emergents (UVE), Aix Marseille Univ, Institut de Recherche pour le Développement (IRD) 190, Institut National de la Santé et de la Recherche Médicale (Inserm) U1207, IHU Méditerranée Infection, 13005 Marseille, France; franck.touret@univ-amu.fr (F.T.); antoine.nougairede@univ-amu.fr (A.N.); xavier.de-lamballerie@univ-amu.fr (X.d.L.); 3 CIRI, Centre International de Recherche en Infectiologie, Team Viral Infection, Metabolism and Immunity, Univ Lyon, Institut National de la Santé et de la Recherche Médicale (Inserm), U1111, Centre National de la Recherche Scientifique (CNRS), UMR5308, Ecole Normale Supérieure de Lyon, Université Claude Bernard Lyon 1, 69007 Lyon, France; clemence.jacquemin@inserm.fr (C.J.); patrice.andre@inserm.fr (P.A.); 4 Unité de Chimie et Biocatalyse, Institut Pasteur, Centre National de la Recherche Scientifique (CNRS), UMR 3523, 28 rue du Dr. Roux, CEDEX 15, 75724 Paris, France; yves.janin@pasteur.fr; 5 Centre d’Etude des Pathologies Respiratoires (CEPR), Institut National de la Santé et de la Recherche Médicale (Inserm), U1100, Faculty of Medecine, University of Tours, 37000 Tours, France; virginie.vasseur@univ-tours.fr (V.V.); mustapha.si-tahar@univ-tours.fr (M.S.-T.); 6 Laboratoire de Santé Animale, Site de Normandie de l’Agence nationale de sécurité sanitaire de l’alimentation, de l’environnement et du travail (ANSES), Physiopathologie et épidémiologie des maladies équines (PhEED) Unit, 14430 Goustranville, France; aymeric.hans@anses.fr (A.H.); jose-carlos.valle-casuso@anses.fr (J.-C.V.-C.)

**Keywords:** SARS-CoV-2, antiviral, chemical screening, DHODH, Vidofludimus, BAY2402234, IPPA-17-A04, NADPH oxidase, Setanaxib

## Abstract

Our therapeutic arsenal against viruses is very limited and the current pandemic of SARS-CoV-2 highlights the critical need for effective antivirals against emerging coronaviruses. Cellular assays allowing a precise quantification of viral replication in high-throughput experimental settings are essential to the screening of chemical libraries and the selection of best antiviral chemical structures. To develop a reporting system for SARS-CoV-2 infection, we generated cell lines expressing a firefly luciferase maintained in an inactive form by a consensus cleavage site for the viral protease 3CL^Pro^ of coronaviruses, so that the luminescent biosensor is turned on upon 3CL^Pro^ expression or SARS-CoV-2 infection. This cellular assay was used to screen a metabolism-oriented library of 492 compounds to identify metabolic vulnerabilities of coronaviruses for developing innovative therapeutic strategies. In agreement with recent reports, inhibitors of pyrimidine biosynthesis were found to prevent SARS-CoV-2 replication. Among the top hits, we also identified the NADPH oxidase (NOX) inhibitor Setanaxib. The anti-SARS-CoV-2 activity of Setanaxib was further confirmed using ACE2-expressing human pulmonary cells Beas2B as well as human primary nasal epithelial cells. Altogether, these results validate our cell-based functional assay and the interest of screening libraries of different origins to identify inhibitors of SARS-CoV-2 for drug repurposing or development.

## 1. Introduction

In January 2020, SARS-CoV-2 was identified as the etiological agent of the current Covid-19 pandemic. Covid-19 is a complex respiratory disease with symptoms ranging from none to severe illness that can lead to death. Thanks to unprecedented worldwide efforts, this virus and the associated diseases are being characterized in great detail and vaccines have been developed and approved in less than a year. Drugs to treat patients with Covid-19 are also urgently needed, but the current therapeutic arsenal is extremely limited despite research endeavors. Many molecules have been evaluated in clinical trials for either treating symptoms of the infection or blocking the virus replication. The anti-inflammatory corticosteroid dexamethasone and the anti-IL6 antibody tocilizumab have been shown to contain the cytokine storm associated with severe Covid19 and to improve patients’ survival in intensive care units [1,2]. Unfortunately, most of the molecules tested so far have shown none (e.g., hydroxychloroquine, interferon or lopinavir/ritonavir) or only minor benefits for patients (e.g., remdesivir, favipiravir). This stresses the persistent need for prophylactic or therapeutic drugs with a clear benefit for patients.

SARS-CoV-2 is a positive-strand RNA virus of the Nidovirales order, Coronaviridae family and Betacoronavirus genus. It is closely related to human coronavirus OC43 (HCoV-OC43) and HKU1 (HCoV-HKU1) which cause common cold, but also to SARS-CoV and MERS-CoV which were respectively responsible for deadly but much more limited pandemics in 2003 and 2012. The development of effective antiviral strategies against coronaviruses should rely first on a better characterization of their replication machinery and interactions with cellular components to identify valuable drug targets. An example of this knowledge-based strategy is the characterization of the virus transcriptional complex that led to the development of S-adenosyl-methionine analogs for blocking the 2′-O-methylation step required for viral mRNA capping [3]. A complementary approach is to conduct phenotypic screenings of chemical libraries using in cellulo infection models without a priori knowledge of the targets. When using libraries containing approved drugs, this may lead to repurposing strategies. Furthermore, hits with a previously well-characterized mode of action may identify cellular pathways and enzymatic reactions required for the virus to replicate. Several screens of approved drug libraries were recently conducted on SARS-CoV-2-infected cultures, leading to the selection of molecules which now require more elaborated in vitro, ex vivo and in vivo model assessments [4,5,6]. Here we report the first screen of a metabolism-oriented library containing well-characterized molecules and approved drugs.

**Figure 1:**
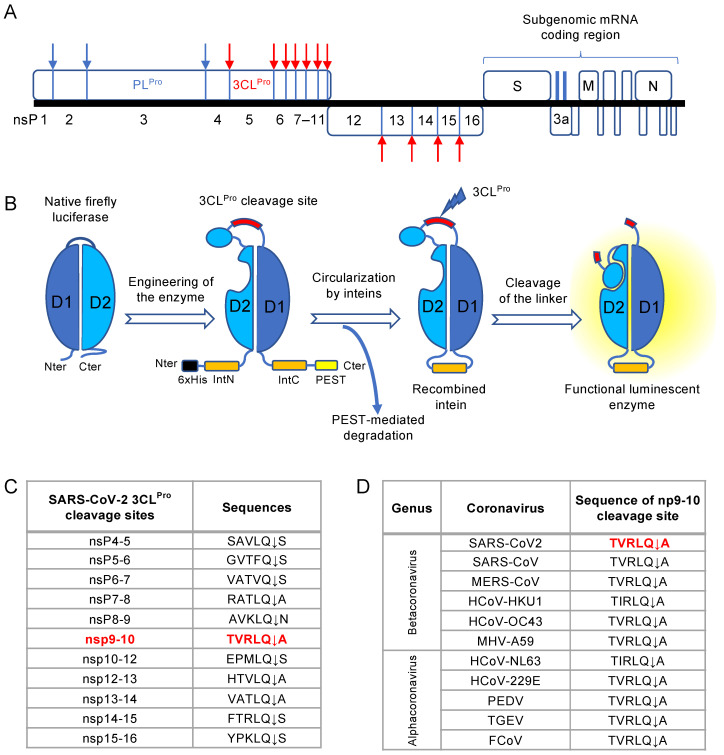
Development of a luminescent biosensor to measure the protease activity of SARS-CoV-2 3CL^Pro^. (**A**) Schematic representation of SARS-CoV-2 genome with corresponding opened reading frames. PL^Pro^ and 3CL^Pro^ cleavage sites within the non-structural polyprotein are indicated by blue and red arrows, respectively. (**B**) Schematic representation of the luminescent biosensor designed for detecting 3CL^Pro^ activity. Firefly luciferase N and C-terminal fragments 4-233 (D1) and 234-544 (D2) were circularly permuted, and linked together by a peptide containing the 3CL^Pro^ cleavage site. The reporter protein is stabilized by circularization thanks to the IntN and IntC domains, while the PEST sequence promotes the degradation of non-ligated proteins. Upon proteolytic cleavage, constrained domains are relaxed and fold into a functional luminescent enzyme. (**C**) List of 3CL^Pro^ cleavage sites that are present in SARS-CoV-2 polyprotein ORF1ab. The nsp9-10 junction that was used in the luminescent biosensor is colored in red. (**D**) Sequence of the nsP9-10 junction across different alpha and betacoronaviruses. MHV is for Murine Hepatitis Virus or murine coronavirus, PEDV is Porcine Epidemic Diarrhea Virus, TGEV is for Transmissible Gastroenteritis Coronavirus and FCoV is for feline coronavirus.

The reported phenotypic screens used to identify SARS-CoV-2 inhibitors essentially rely on host cell viability as an indicator of viral growth [4,5,6], assessing the inhibition of the virus cytopathic effect after multicyclic replication at relatively late timepoints. More direct measures of viral infection by immunostaining and fluorescence imaging have been used but are often more challenging to implement in a format suitable for testing hundreds of molecules and require automated microscopes for data acquisition [7,8]. Interestingly, the SARS-CoV-2 genome encodes a 3C-like protease (3CL^Pro^; nsP5) that is responsible for the cis- and trans-processing of the viral polyprotein and the destruction of host protein targets (Figure 1A). This proteolytic activity of 3CL^Pro^ could thus be used as a marker of SARS-CoV-2 replication as previously reported for the NS3/4A protease of HCV [9]. Furthermore, the target sequence for the 3CL^Pro^ protease is highly conserved among coronaviruses, opening the possibility for a versatile assay compatible with multiple coronaviruses.

To establish a bioluminescent reporter system and measure the activity of 3CL^Pro^ in infected cells, two groups have described a circularly permuted firefly luciferase constrained by an engineered site corresponding to the 3CL^Pro^ target sequence [10,11,12]. This cleavage site maintains the recombinant luciferase in an inactive, constrained form (Figure 1B). When processed by 3CL^Pro^, the two fragments of the luciferase are relaxed and the bioluminescent activity is recovered. This luminescent biosensor was validated by transient overexpression of 3CL^Pro^ from different coronaviruses, but was never used to monitor viral infection. Here, we report the development of an improved luminescent biosensor and reporter cell line based on this principle to identify antiviral molecules by monitoring the 3CL^Pro^ activity of SARS-CoV-2. This assay is compatible with the screening of large chemical libraries which requires miniaturized and automated in vitro infection models. Moreover, the replication of viruses critically depends on the fine tuning of their host metabolic pathways which could thus represent potential targets for a host-directed antiviral approach [13]. Accordingly, this new reporter assay was used to screen a focused library of 492 drugs specifically targeting cellular metabolic pathways. Results of this screening and additional studies are presented here with a special focus on Setanaxib (GKT137831; [14]), a NADPH oxidase 1 and 4 (NOX1/4) inhibitor in clinical trial, that could be repositioned rapidly against SARS-CoV-2 infection.

## 2. Materials and Methods

### 2.1. Cells and Reagents

Vero E6 (ATCC CRL-1586), Caco-2 (ATCC HTB-37), Huh7 (a gift from Marco Binder; Heidelberg University, Heidelberg, Germany), Huh7-Lunet (a gift from Ralph Bartenschlager; Heidelberg University, Heidelberg, Germany) and Huh7.5 (a gift fom Paul D. Olivo; Apath LLC, New York City, NY, USA) cells were grown in DMEM with GlutaMAX (10566016; Thermo Fisher Scientific, Waltham, MA, USA) supplemented with 10% fetal calf serum (FCS) and penicillin/streptomycin. Beas-2B cells were grown in F-12K supplemented with 10% FCS, 1% L-glutamine, 10 mM HEPES, and penicillin/streptomycin. Beas-2B cells expressing ACE2 were obtained by transduction with the lentiviral vector RRL.sin.cPPT.SFFV/Ace2.IRES-hygro.WPRE, a gift from Dr. Caroline Goujon (Addgene plasmid #145839) followed by hygromycin selection [15]. Culture medium and additives were from ThermoFisher/Gibco except FCS (Dutcher). Setanaxib was purchased from MedChemExpress (Monmouth Junction, NJ, USA). Hydroxychloroquine sulfate was from Sigma-Aldrich (St Louis, MO, USA). Racemic BAY2402234 was prepared following the synthesis described in a patent by Hassfeld, J. & al [16] with the difference that racemic trifluoro isopropanol was used instead of the S enantiomer.

### 2.2. Virus Strains

The recombinant NeonGreen SARS-CoV-2 virus (icSARS-CoV-2-mNG) was obtained by introducing the mNeonGreen reporter gene into ORF7 of the viral genome as described elsewhere [17]. SARS-CoV-2 strain 2019-nCoV/USA_WA1/2020 was isolated by the CDC in the United States, from the first patient diagnosed in the US. For stock production, Vero E6 cells were infected in DMEM (MOI = 0.01). After 90 min incubation at 37 °C, medium was replaced with DMEM-2% FBS and cells were incubated for two days. Viral supernatant was collected and centrifuged (400× *g*, 5 min), and aliquoted for being stored at −80 °C. Virus was titrated in plaque forming unit by classic dilution limit assay. SARS-CoV-2 strain BavPat1 used in Supplementary Figure S2 was obtained from Pr. C. Drosten through EVA GLOBAL (accessed on 13 February 2020; https://www.european-virus-archive.com/). For stock production, confluent Vero E6 cells growing in MEM-2.5% FCS was inoculated at MOI 0.001. Cell supernatant medium was harvested at the peak of replication, centrifuged and supplemented with 25 mM HEPES (Sigma-Aldrich, St Louis, MO, USA) before being stored frozen in aliquots at −80 °C. Virus was titrated by classic TCID_50_ assay. All experiments with infectious SARS-CoV-2 were conducted in a biosafety level 3 laboratory. Measles virus Edmonston strain was amplified on Vero E6 cells and titrated by TCID_50_.

### 2.3. Establishing Reporter Cell Lines

The luciferase biosensor for 3CL^Pro^ activity was based on CycLuc_TEVS, a circularly permuted luciferase described by Fink T et al. [18]. The CycLuc_TEVS luciferase contains circularly permuted domains 4-233 and 234-544 of firefly luciferase separated by a TEV cleavage site and flanked by a 6xHIS tag, IntN and IntC domains of the NpuDnaE intein, and a PEST degradation sequence (Figure 1B). To replace the TEV cleavage site by a 3CL^Pro^ cleavage site (STVRLQ↓AGTATE), the DNA sequence of CycLuc_TEVS (a gift from Roman Jerala; Addgene #119207) was modified by replacing the ggatccgaaaatctctatttccagagcggcggt sequence with tctacagttagattgcaagctggaactgctactgaa. The corresponding CycLuc_3CL^Pro^ DNA sequence was obtained by gene synthesis from GeneArt (Thermo Fisher Scientific, Waltham, MA, USA) and cloned in pDONR221 of the Gateway system (Thermo Fisher Scientific, Waltham, MA, USA). The sequence was shuffled by in vitro recombination into the Gateway-compatible lentiviral expression vector pLEX_307 (a gift from David Root; Addgene #41392; Addgene, Watertown, MA, USA). Lentiviral particles were produced and applied to target cells following manufacturer’s recommendations. Transduced cells were then selected for at least 7 days in culture medium supplemented with puromycin (1 μg/mL). Cells were stably transduced as no loss of the reporter construct was observed after one month of culture corresponding to at least ten passages in the absence of selection antibiotic.

To assess the activation of the CycloLuc_3CL^Pro^ reporter, reporter cells were transfected with pmCherry-C1 nsP5 (SARS-CoV-2), a plasmid expressing 3CL^Pro^ from SARS-CoV-2. This construct was obtained by Gateway cloning using pDONR223 SARS-CoV-2 NSP5 as donor vector (a gift from Fritz Roth; Addgene #141259; Addgene, Watertown, MA, USA) and pmCherry-C1-GW as destination vector [19,20]. Cells were transfected with JetPrime reagent following manufacturer’s recommendations (Polyplus-transfection, Illkirch-Graffenstaden, France). Briefly, 60 × 10^3^ cells were plated in a 48-well plate and transfected one day later with 0.75 μg of pmCherry-C1 nsP5 (SARS-CoV-2) or pmCherry-C1-GW as a control. After 24 h of culture, cells were harvested and resuspended in 800 μL of culture medium. To quantify luciferase activity, 100 μL of cell suspension was dispensed in a white, flat-bottom 96-well plate and 50 μL of Bright-Glo Luciferase assay reagent (Promega, Madison, WI, USA) was added. After 5 min of incubation at room temperature, luminescence was quantified with a Mithras luminometer (Berthold Technologies, Bad Wildbad, Germany).

### 2.4. Screening Procedure

The 492 compounds selected for the screen were from the Metabolism-related Compound Library of APExBio (L1032; DiscoveryProbe; APExBio Technology, Houston, TX, USA) which was purchased from Stratech (Ely, UK) and was stored at −80 °C. Stock solutions provided by the manufacturer were at 10 mM in DMSO unless specified otherwise. To generate screening plates, 2 μL from stock solutions were dispensed in white, flat-bottom 96-well screening plates (Greiner 655083; Greiner, Kremsmünster, Austria). 200 μL of DMEM-10% FCS + PS were added to each well and after proper mixing, 100 μL were transferred into an empty plate in order to obtain duplicates of each screening plate. Huh7.5 cells with the CycLuc_3CL^Pro^ reporter (Huh7.5_ CycLuc_3CL^Pro^) were resuspended in DMEM-10% FCS + PS at 2 × 10^5^ cells/mL, and dispensed under 100 μL corresponding to 2 × 10^4^ cells/well. All drugs were thus tested at a final concentration of 50 μM. The 7 control wells (column 12) were treated with DMSO alone. After 24 h of culture, the first set of screening plates was infected by adding 20 μL of SARS-CoV-2 stock solution in culture medium (MOI = 0.5). Only 4 of the control wells infected (positive controls), whereas 3 control wells were left uninfected (negative controls). The second set of screening plates was left uninfected to determine the impact of each drug on expression of the CycLuc_3CL^Pro^ reporter. 

After 24 h of culture, 100 μL of culture supernatant was removed and 50 μL of Bright-Glo Luciferase assay reagent (Promega, Madison, WI, USA) was added. After 5 min of incubation at room temperature, luminescence was quantified with a Tecan Spark 10M (infected cells) or a Mithras luminometer from Berthold (uninfected cells). In parallel and to evaluate directly the cytotoxicity of drugs, Huh7 cells at 8 × 10^3^ cells/well were treated with drugs from the library at 50 μM in 200 μL of culture medium. After 3 days of culture, 100 μL of culture supernatant was removed and 50 μL of CellTiter-Glo Luminescent Cell Viability reagent (Promega, Madison, WI, USA) was added. After 10 min of incubation at room temperature, luminescence was quantified with a Tecan M200 luminometer.

For each plate, raw luminescent values were first normalized to the average of non-infected control wells (μ−) to eliminate plate-to-plate variations. The Z′ factor of the assay was calculated from means (μ+ and μ−) and standard deviations (σ+ and σ−) of negative and positive controls such as Z′ = 1−3 × (σ+ + σ−)/(μ+−μ−) [21]. The signal-to-background ratio (S/B ratio) corresponds to μ+/μ−. For each drug, the normalized luminescence ratio (NLR) of infected over non-infected cultures was calculated to take into account the potential impact of the drug on the expression of the CycLuc_3CL^Pro^ reporter protein. Finally, the NLR value obtained for each drug was used to express results as percentage of inhibition when compared to DMSO-treatment.

### 2.5. Inhibition of Viral Replication

For data presented in Section 3.4, 1 × 10^4^ Huh7.5 cells or 2 × 10^4^ Beas-2B-ACE2 cells were seeded in 100 µL of culture medium in 96-well plates one day prior to infection. The next day, 100 μL of 2× concentrated Setanaxib was added to obtain a final concentration of 25 μM. Control wells were supplemented with DMSO. After an additional 24 h of culture, cells were infected with SARS-CoV-2 expressing mNeonGreen. 20 µL of a virus mix diluted in medium was added to the wells at the correct MOI. Plates were incubated for 24 h at 37 °C after which 100 µL of the supernatant was collected for viral RNA purification and mNeonGreen expression was determined by fluorescence microscopy. Viral RNA was quantified by RT-qPCR as previously described using primers and probe indicated in Table 1 [22]. For the evaluation of Setanaxib toxicity, the same culture conditions were set without addition of the virus, and cell viability was measured using CellTiter-Glo reagent (Promega). The CellTiter-Glo was added following the manufacturer’s instructions and luminescence was determined with a Tecan M200 luminometer.

For data presented in Supplementary Figure S2, 5 × 10^4^ Vero E6 cells were seeded in 100 µL of MEM-2.5% FCS in 96-well plates. The next day, eight 2-fold serial dilutions of compounds (depends on compound), in triplicates were added to the cells (25 µL/well, in 2.5% FCS-containing medium). Four virus control wells (per virus) were supplemented with 25 µL medium and four cell control wells were supplemented with 50 µL of medium. After 15 min, 25 µL of a virus mix diluted in medium was added to the wells at the correct MOI (0.002), determined so that the replication growth is still in the log growth curve for the readout at day 2. Plates were incubated for 2 days at 37 °C after which 100 µL of the supernatant was collected for viral RNA purification. Viral RNA was quantified by RT-qPCR as previously described using primers and probe indicated in Table 2 [4]. For the evaluation of the CC_50_ (the concentration that reduces the total cell number by 50%), the same culture conditions were set as for the determination of the EC_50_ and EC_90_ (the concentrations that reduce the viral load by 50% and 90%, respectively), without addition of the virus, and cell viability was measured using CellTiter-Blue reagent (Promega). The CellTiter-Blue was added following the manufacturer’s instructions. Plates were incubated for 2 h prior recording fluorescence (560/590 nm) with a Tecan Infinite 200Pro machine.

### 2.6. Culture and Infection of Human Primary Nasal Epithelial Cells (PNECs)

Primary human nasal epithelial cells were purchased from Epithelix (Plan-les-Ouates, Switzerland) and cultured following previously described procedure [23]. After thawing and centrifugation, cells were recovered in cold Basement Membrane Extract (BME; Cultrex Reduced Growth Factor BME Type 2, Pathclear, 3533-010-02; Bio-techne, Minneapolis, MN, USA). Three droplets of 30 μL were dispensed in 12-well plates, and then incubated for 1 h at 37 °C to induce polymerization before adding organoid medium containing Advanced DMEM/F12 (1X), Hepes (10 mM), GlutaMax (1X), Nicotinamide (5 mM), N-acetylcysteine (1.25 mM), B27 supplement (1X), SB202190 (500 nM), Y-27632 (5 μM), A83-01 (500 nM), Noggin (100 ng/mL), FGF 10 (100 ng/mL), FGF 7 (25 ng/mL) and R-Spondin 1 (500 ng/mL). Medium was refreshed twice a week for 1–2 week. Organoids were then passaged. Culture medium was removed and organoids were resuspended in cold PBS to dissolve BME. After centrifugation, organoids were dissociated in Trypsin-Versene EDTA (Lonza, Basel, Switzerland) and trypsin was neutralized with SBTI. Cells were resuspended in BME as above at 100,000 cells/droplet and cultured in organoid medium for an additional 1–2 weeks. Organoids were harvested as above and 8 × 10^6^ cells were plated in a pre-coated T75 flask (coating solution: PBS 1X; Fibronectin 5 μg/mL; PureCol 30 μg/mL; BSA 10 μg/mL) containing Complete PneumaCult™-Ex Medium (Complete PneumaCult™-Ex Medium, PneumaCult™-Ex Supplement, Hydrocortisone and Primocin; StemCell Technologies, Vancouver, Canada). After 4 days, cells were harvested using soft-trypsin, and dispensed at 30,000 cells/well in pre-coated 96-well plates. One day later, culture medium was removed and replaced with 100 μL of infection medium (50/50 Advanced DMEM/F12 (#11540446) and BEGM (#CC-3170), Primocin, HEPES, and GlutaMax). Setanaxib was added at 12.5 or 25 μM, and cells were infected with SARS-CoV-2 expressing mNeonGreen (MOI = 0.7). Viral infection was determined 24 h later by fluorescence microscopy.

### 2.7. Cell-to-Cell Fusion Assay

The β-galactosidase complementation-based fusion assay was performed as previously described [24]. Briefly, HEK-293T cells were seeded in two T25 flasks. The next day, cells were transfected using Transit LT1 (Mirus Bio, Madison, WI, USA) and OptiMEM (Gibco, Thermo Fisher Scientific, Waltham, MA, USA). The first flask was transfected with 3 μg of pCAGGS-Spike-WT + 1.7 μg of pCAGGS-Neo-α-β-gal + 0.33 μg of pCSCMV-tdTomato; the second flask was transfected with 3 μg of pCAGGS-puro-ACE2-Venus + 1.7 μg of pCAGGS-Neo-Ω-β-gal. At 7 h post transfection, cells expressing the Spike protein were seeded in black 96-well plates (Corning, Corning, NY, USA) coated with poly-D-Lysine (Sigma-Aldrich, St Louis, MO, USA). Cells were incubated for 15 h at 37 °C to allow attachment, and Setanaxib was added to cultures. Next, cells expressing ACE2 were deposited, and plates were incubated for an additional 5 h to allow cell-to-cell fusion at 37 °C. β-galactosidase activity was determined in culture wells using the Galacto-Star™ β-Galactosidase Reporter Gene Assay System for Mammalian Cells (Thermo Fisher Scientific, Waltham, MA, USA) following manufacturer’s recommendations. Luminescence was measured after 45 min of incubation with a Tecan M200 luminometer.

### 2.8. Lysosomal Staining

Vero cells were seeded in 24-well plates at 40,000 cells/well in 500 μL of culture medium. After 24 h, cells were treated with Setanaxib (25 μM), hydroxychloroquine (50 μM) or DMSO alone in a final volume of 1 mL. After 4 h at 37 °C, LysoTracker Deep Red (L12492—Invitrogen, Thermo Fisher Scientific, Waltham, MA, USA) was added at 50 nM (final volume: 1.5 mL) and cells were incubated for 30 min at 37 °C. Cells were washed with 1.5 mL of HBSS (Gibco 14025-050, Thermo Fisher Scientific, Waltham, MA, USA) three times, and lysosomal staining was immediately observed by fluorescence microscopy.

## 3. Results

### 3.1. Design of a Luciferase-Based Biosensor for the Detection of 3CL^Pro^ Activity

To develop a bioluminescence-based biosensor of the proteolytic activity of SARS-CoV-2 3CL^Pro^, we took advantage of a previously reported construct based on circularly permuted N- and C-terminal fragments of firefly luciferase linked by a protease cleavage site (Figure 1B) [18]. This permutation locks the luciferase in the opened conformation, and prevents luciferin processing [25,26]. When the linker is cleaved, conformational transition to the closed conformation is permitted and the luciferase activity is restored. In addition, the biosensor is stabilized by cyclization using N and C-terminal intein domains, IntN and IntC, which catalyze head-to-tail linkage. This type of circularly-permuted, cyclized luciferase was successfully used to monitor the protease activity of 3CL^Pro^ from Porcine Epidemic Diarrhea Virus (PEDV), a member of the Alphacoronavirus genus [10]. In addition, to force the elimination of reporter proteins that failed to cyclize and potentially generate background signal, a PEST degradation sequence was added at the C-terminus of the reporter construct [18]. Finally, the cleavage site inserted in the biosensor protein was carefully selected for an efficient processing by 3CL^Pro^ from SARS-CoV-2. The optimal consensus sequence for the SARS-CoV 3CL^Pro^ substrate was previously reported by Chuck CP. et al. and is V/F/T-C/V-R/V-L-Q↓S/A/C [27]. We thus analyzed the 11 cleavage sites of 3CL^Pro^ within the polyprotein coded by SARS-CoV-2 (Figure 1C) and identified a perfect match with this consensus at the nsP9-nsP10 junction (TVRLQ↓A). In addition, this cleavage site is highly conserved across human and animal coronaviruses (Figure 1D). We thus decided to introduce this sequence as the cleavable linker in our luciferase biosensor.

### 3.2. Validation of the 3CL^Pro^ Biosensor

**Figure 2:**
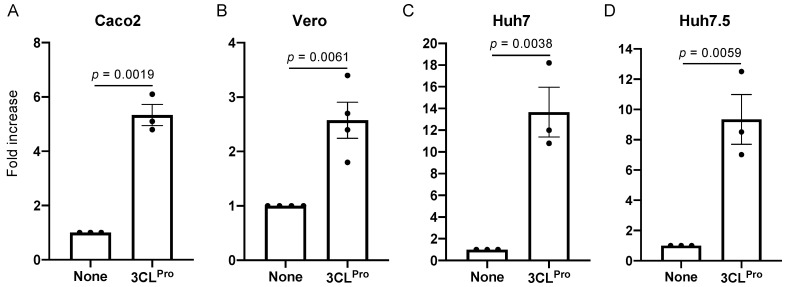
Activation of the luminescent biosensor by SARS-CoV-2 3CL^Pro^ expression in different cell lines. (**A**–**D**) Caco-2, E6 Vero, Huh7 and Huh7.5 cells were stably transfected with a lentiviral vector to constitutively express the 3CL^Pro^ luminescent biosensor. Stable cell lines were transfected with an expression plasmid that was either empty (None) or encoding SARS-CoV-2 3CL^Pro^. After 24 h of culture, luciferase activity in culture wells was determined. Data correspond to means ± SEM from 3 independent experiments in triplicate and *p* values were determined by Student’s *t*-test.

The coding sequence of the biosensor defined above was obtained by gene synthesis and cloned into a lentiviral vector suitable for gene transduction and antibiotic selection. Lentiviral particles were produced and used for transducing four different cell lines which are permissive to SARS-CoV-2. This included Caco-2 (human colorectal adenocarcinoma), E6 Vero (kidney epithelial cells from African green monkey), Huh7 (human hepatocellular carcinoma) and Huh7.5 (a subclone of Huh7 defective for the innate immune response because of a mutation in RIG-I). After one week of antibiotic selection to ensure that transgene constructs were stably integrated, cells were transfected with a plasmid encoding SARS-CoV-2 3CL^Pro^ or an empty plasmid as a control. Following 24 h of culture, the luciferase activity was measured in transfected cells. A significant increase in bioluminescence was observed in the presence of 3CL^Pro^ in all four reporter cells (Figure 2A–D). The induction was however limited in Vero cells most likely because of a much higher background signal (Figure 2B) reflecting the presence of an endogenous protease constitutively activating the luminescent biosensor.

**Figure 3:**
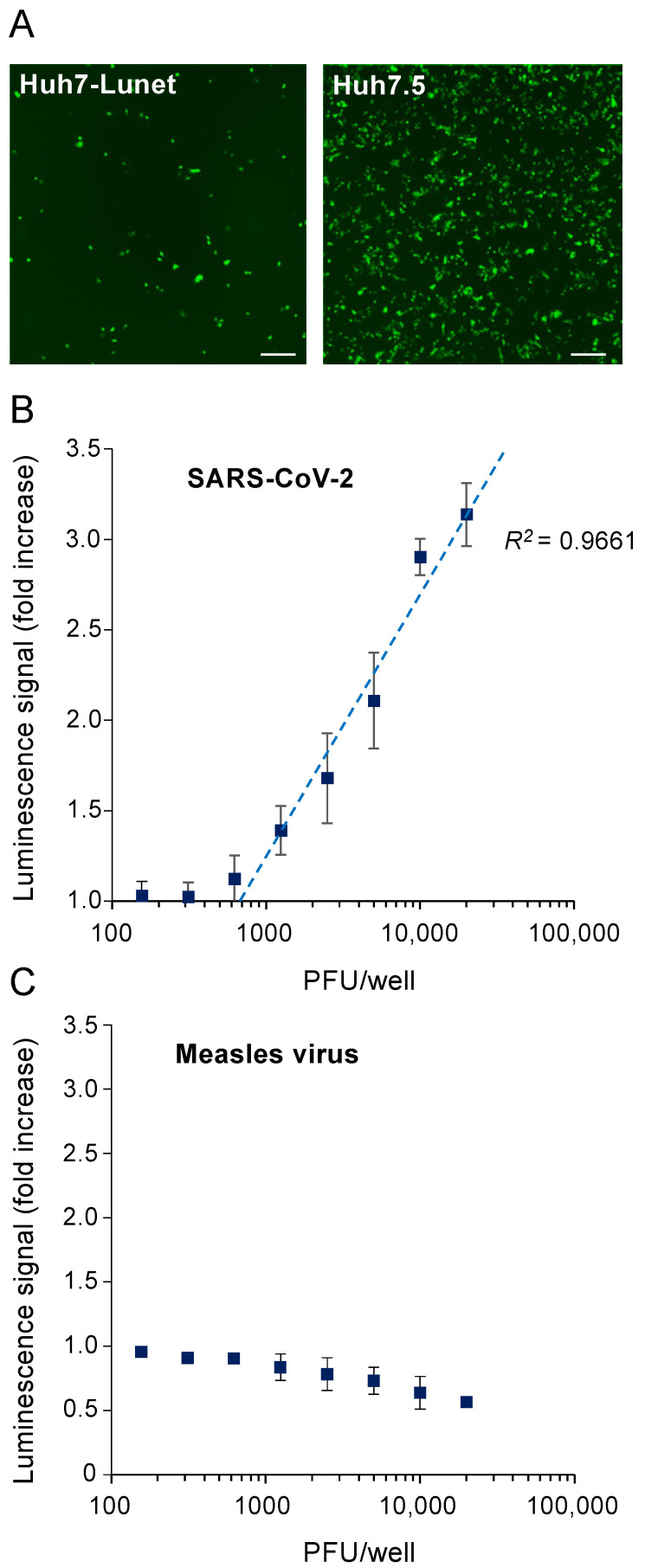
Activation of the luminescent biosensor by SARS-CoV-2 infection. (**A**) To compare their susceptibility to infection, Huh7-Lunet and Huh7.5 cells were infected with a recombinant strain of SARS-CoV-2 expressing mNeonGreen as a reporter (MOI = 0.5). After 20 h of culture, infection was determined by fluorescence microscopy and representative images are presented. Scale bar is 300 μm. (**B**) In 96-well plates, Huh7.5 cells stably transfected with the 3CL^Pro^ biosensor were infected with increasing amounts of SARS-CoV-2 (strain 2019-nCoV/USA_WA1/2020) ranging from 156 to 20,000 PFUs/well (i.e., 0.004 to 0.5 MOIs). After 24 h of culture, luciferase activity was determined. Data correspond to means ± SEM from 3 independent experiments in triplicate (*n* = 1) or in duplicate (*n* = 2). Linear regression was determined for PFUs/well ranging from 625 to 20,000 (i.e., 0.0156 to 0.5 MOIs) where some luciferase signal was detected. (**C**) Same experiment as in (**B**) but cells were infected with measles virus. Data correspond to means ± SEM from six experiments.

We then determined whether SARS-CoV-2 infection was able to activate the luminescent biosensor. The Huh7.5 cells were used since they are easier to grow than Caco-2 cells and because of their higher permissiveness to SARS-CoV-2 compared to the parental cells Huh7 [8]. Their high susceptibility to infection was confirmed using a recombinant virus expressing the fluorescent protein mNeonGreen (Figure 3A). In comparison, the Huh7-Lunet cells that are highly permissive to Hepatitis C virus alike Huh7.5 cells poorly replicated SARS-CoV-2 [28]. The Huh7.5 cells were thus infected with increasing amounts of SARS-CoV-2 infectious particles corresponding to multiplicities of infections (MOI) ranging from 0.004 to 0.5. After 24 h of culture, the luciferase activity was determined in infected cells and activation of the 3CL^Pro^ biosensor was linearly proportional to the logarithm of MOI (Figure 3B). This time point was selected to get the highest luminescence related to infection in the absence of viral cytopathic effect. As a control, we checked that a virus without cysteine protease such as measles virus (MeV) did not activate this bioluminescent biosensor (Figure 3C). This validated the sensitivity and specificity of the reporter system for a SARS-CoV-2 infection at such an early time point.

### 3.3. Screening of a Metabolism-Oriented Compounds Library

We then tested if our cell-based biosensor was suitable for high-throughput screening of small molecules. Viruses in general, and specifically coronaviruses, are known for hijacking metabolic pathways through specific interactions to fulfill their needs in energy and metabolites. Pharmacological modulators of cell metabolism thus have a plausible potential as antivirals by interfering with the metabolic reprogramming triggered by viruses. To establish a proof of concept, we thus selected a set of 492 molecules inhibiting or activating metabolic pathways which were assayed in 96-well plates at 50 μM using the Huh7.5 cells expressing the 3CL^Pro^ biosensor. After 24 h of culture, cells were infected with SARS-CoV-2 (MOI = 0.5), and cultured for an additional 24 h before measuring luciferase activity. Each plate included four positive and three negative controls corresponding to infected and non-infected cells, respectively. 

**Figure 4:**
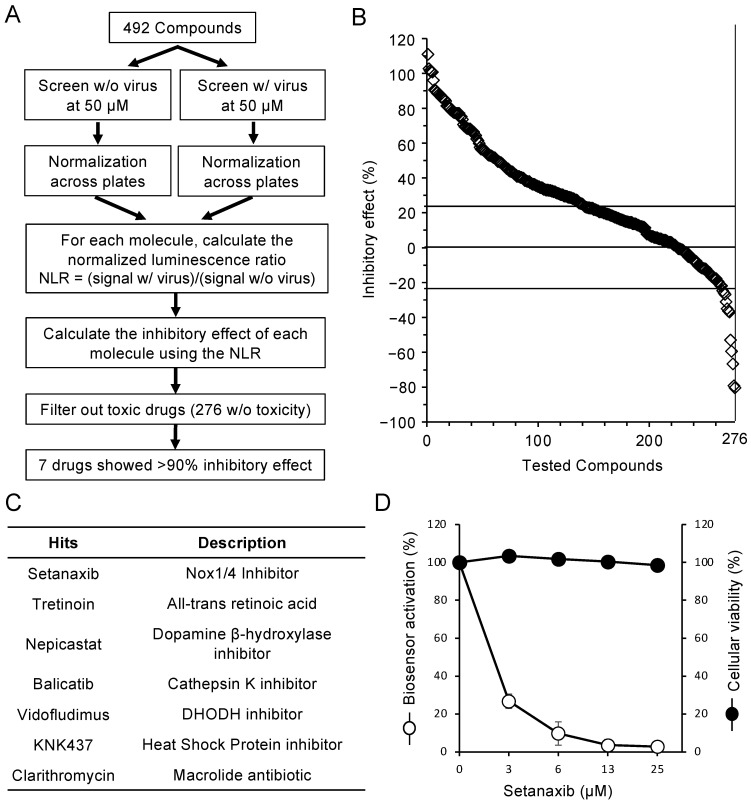
Identification of SARS-CoV-2 inhibitors by screening a chemical library of metabolic modulators. (**A**) Filtering pipeline for the selection of hit compounds. A total of 492 compounds were tested at 50 μM on Huh7.5 expressing the luminescent biosensor for 3CL^Pro^. 20,000 cells per well were treated with the drugs for 24 h and then infected with SARS-CoV-2 (strain 2019-nCoV/USA_WA1/2020; MOI = 0.5; “screen w/ virus”) or were left uninfected (“screen w/o virus”). After 24 h, luciferase activity was determined and results were normalized across plates using DMSO-treated, uninfected control wells as reference. Activation of the luminescent biosensor upon SARS-CoV-2 infection was determined by calculating the normalized luminescence ratio (NLR = “luminescence w/ virus” over “luminescence w/o virus”), and results were expressed as a percentage of inhibitory effect compared to the DMSO-treated, infected control wells. Compounds showing no sign of cellular toxicity (see Section 2.4 for details) and inhibiting by 90% or more the activation of the luminescent biosensor in SARS-CoV-2 infected wells were selected. (**B**) Inhibitory effect of the 276 compounds showing no sign of cellular toxicity. Those with a negative inhibitory effect actually increased the activation of the luminescent biosensor upon SARS-CoV-2 infection. (**C**) List of the molecules selected from the screen. (**D**) Inhibitory effect of Setanaxib on the activation of the 3CL^Pro^ biosensor by SARS-CoV-2. Experiment was conducted as in (**A**) but with increasing concentrations of Setanaxib and results were expressed as percentage of activity compared to the infected, untreated control. Data correspond to means ± SEM from one experiment in triplicate.

We established an algorithm to analyze data and identify potential inhibitors of SARS-CoV-2 (Figure 4A). Raw luminescence data were normalized using the mean of internal negative controls to eliminate plate-to-plate variations. Across the six screening plates, the signal-to-background (S/B) ratio between infected and non-infected control wells was on average 2.86. We also calculated the Z′ factor which reflects the capacity of the assay to discriminate positive controls (infected wells) from negative controls (non-infected wells) (see Section 2.4). The Z′ factor was on average 0.54 across the six screening plates, which is above the threshold of 0.5 characterizing an excellent assay and thus validates the screen [21]. Because some drugs alone could have a significant impact on the background activity of the luciferase biosensor, we performed a control experiment without infecting the cells. For each drug, we calculated a normalized luminescence ratio (NLR) corresponding to the signal in infected over non-infected cultures. Using this NLR value, we calculated the percentage of inhibitory effect of the drug when compared to DMSO treatment.

Some drugs decreased luciferase activity in the absence of infection, suggesting some detrimental effect on the expression of the biosensor. We thus discarded 158 drugs that decreased the luminescence signal in non-infected cells by more than 30%. In addition, we had previously tested as part of an independent research project all 492 drugs on the long-term proliferation and survival of Huh7 cells by using ATP level in culture wells as a measure of cellular viability (CellTiter-Glo reagent; Promega). Drugs that reduced cellular proliferation or viability by more than 30% in this secondary assay were also discarded (191 drugs). Interestingly, 84% of the drugs reducing the expression of the biosensor in the absence of infection also reduced cellular viability, thus highlighting the link existing between these two filtering criteria. In total, 276 drugs passed these toxicity filters (Figure 4B, Supplementary Table S1). Finally, we selected molecules that inhibited by more than 90% the induction the luciferase biosensor by SARS-CoV-2. This led to 7 molecules which passed these stringent criteria, and these were further studied (Figure 4C, Supplementary Figure S1).

Vidofludimus, an inhibitor of pyrimidine biosynthesis targeting dihydroorotate dehydrogenase (DHODH), which was recently reported to inhibit SARS-CoV-2 replication, was in the list of identified hits [29]. To validate the antiviral activity of this drug in a different cell line, Vidofludimus was assessed on Vero E6 cells, which is commonly used as an in vitro infection model for SARS-CoV-2. Cells were treated with increasing concentrations of Vidofludimus and then infected with SARS-CoV-2 at a MOI of 0.002. After 48 h of culture, supernatants were harvested and the production of viral particles in culture supernatants was determined by RT-qPCR. As shown in Supplementary Figure S2A, Vidofludimus inhibited the production of SARS-CoV-2 as recently reported [29]. EC50 and 90 values reached 3.2 and 11.4 μM, respectively. Other well-known DHODH inhibitors such as IPPA17-A04 [30] or the racemic form of BAY2402234 [31] also blocked the production of SARS-CoV-2 particles (Supplementary Figure S2B). In addition, the antiviral effect of IPPA17-A04 was reverted by the addition of uridine, thus confirming the causative link between pyrimidine depletion and SARS-CoV-2 inhibition in cells treated with DHODH inhibitors (Supplementary Figure S2B). Tretinoin, a retinoic acid receptor (RAR) agonist that was suggested to block the ion channel formed by SARS-CoV-2 envelope (E) protein [32], and clarithromycin, a macrolide antibiotic closely related to azithromycin which blocks coronavirus replication in vitro [4], were also in this short list of hit compounds. Altogether, this validated our selection strategy although DHODH inhibitors have yet to be shown as useful frontline antivirals in clinic.

In any case, the most effective molecule in our list was Setanaxib (GKT137831), a NADPH oxidases 1 and 4 (NOX1/4), which also scored as a hit in a recently published SARS-CoV-2 inhibitors screen [6]. When evaluated at different concentrations in our assay, results showed that a 6 μM concentration of Setanaxib inhibited by 90% the SARS-CoV-2 activation of the luciferase biosensor by SARS-CoV-2 without toxicity (Figure 4D). Because Setanaxib is already in clinical trial for other indications, we thus investigated in details SARS-CoV-2 inhibition by this molecule.

### 3.4. Inhibition of SARS-CoV-2 Replication by Setanaxib

**Figure 5:**
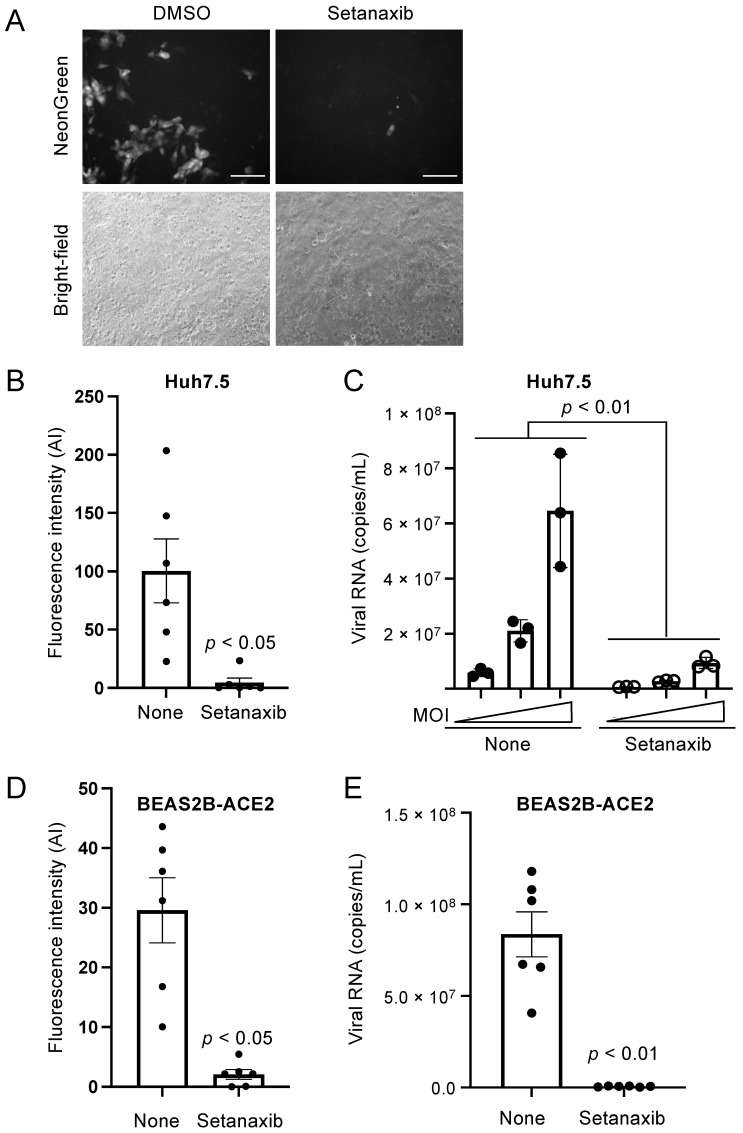
Inhibition of SARS-CoV-2 replication by Setanaxib. (**A**,**B**) Huh7.5 cells were treated for 24 h with Setanaxib (25 μM) or left untreated, and then infected with recombinant SARS-CoV-2 expressing mNeonGreen (MOI = 0.1). After 24 h of culture, infection was determined by fluorescence microscopy. A representative image is presented in (**A**). Scale bar is 100 μm. A total of six randomly selected microscopy fields from three independent culture wells (one experiment in triplicate) were analyzed for mNeonGreen expression. Fluorescence quantification was performed with ImageJ, and corresponding data are presented in (**B**). (**C**) Same as in (**A**) but cells were infected with recombinant SARS-CoV-2 expressing mNeonGreen at 0.004, 0.02 and 0.1 MOIs. After 24 h of culture, supernatants were harvested and SARS-CoV-2 RNAs were quantified by RT-qPCR. (**D**) Beas-2B cells expressing ACE2 were infected with SARS-CoV-2 expressing mNeonGreen (MOI = 0.004) and cultured without or with Setanaxib at 25 μM. After 48 h, infection was determined by fluorescence microscopy for mNeonGreen expression. Quantitative data correspond to the fluorescence signal from six independent culture wells obtained with ImageJ (two independent experiments in triplicate). (**E**) Same experiment as in (**D**) but culture supernatants were collected to quantify viral RNA by RT-qPCR. Data correspond to means ± SEM. Statistical significance was determined by Student’s *t*-test using Prism (GraphPad Software, San Diego, CA, USA) for all panels except (**C**) where two-way ANOVA was used.

The antiviral effect of Setanaxib was first assayed in Huh7.5 cells infected with a recombinant strain of SARS-CoV-2 expressing the fluorescent protein mNeonGreen as reporter (MOI = 0.1). After 24 h of culture, fluorescence microscopy confirmed the inhibition of viral infection in these cells (Figure 5A,B). In addition, viral RNAs present in culture supernatants were measured by RT-qPCR to evaluate the release of virus. As shown in Figure 5C, Setanaxib strongly inhibited the production of viral particles from Huh7.5 cultures infected at different MOIs (0.004; 0.02; 0.1). To validate the antiviral activity of this drug in a different cell line, we also used human lung epithelial cells BEAS-2B which had been transduced with SARS-CoV-2 receptor ACE2 (Angiotensin-Converting Enzyme 2). The cells were treated with Setanaxib and immediately infected with SARS-CoV-2 expressing mNeonGreen at a MOI of 0.004. After 48 h of culture, fluorescence microscopy confirmed the inhibition of viral infection by Setanaxib (Figure 5D). The level of viral RNAs present in culture supernatants was also quantified by RT-qPCR and this confirmed a strong viral production inhibition by Setanaxib (Figure 5E).

**Figure 6:**
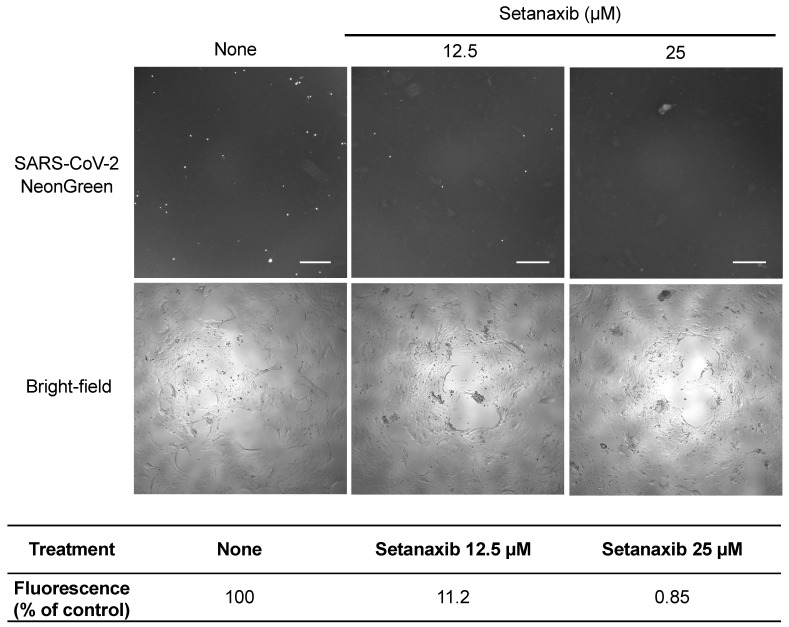
Setanaxib is inhibiting SARS-CoV-2 infection in human primary nasal epithelial cells. Primary nasal epithelial cells from healthy donors were infected with SARS-CoV-2 expressing mNeonGreen (MOI = 0.7) and cultured without or with Setanaxib at 12.5 or 25 μM. After 24 h, infection was determined by fluorescence microscopy for mNeonGreen expression. Scale bar is 300 μm. Fluorescence signal from culture wells was determined with ImageJ. Results are expressed as percentage of the untreated control well.

Finally, the antiviral activity of Setanaxib was determined on human primary nasal epithelial cells (PNECs) from a healthy donor. Cultures were infected by SARS-CoV-2 expressing mNeonGreen (MOI = 0.7) with or without Setanaxib at 12.5 or 25 μM. After 24 h of culture, viral infection was determined by fluorescence microscopy (Figure 6; upper panel). Fluorescence quantification in each culture well showed a dose-dependent inhibition of viral growth (Figure 6; lower panel). Altogether, these results support the inhibition of SARS-CoV-2 by Setanaxib in different cellular models including human primary cells from the respiratory tract.

### 3.5. Setanaxib Does Not Affect 3CL^Pro^ Activity or Viral Fusion

**Figure 7:**
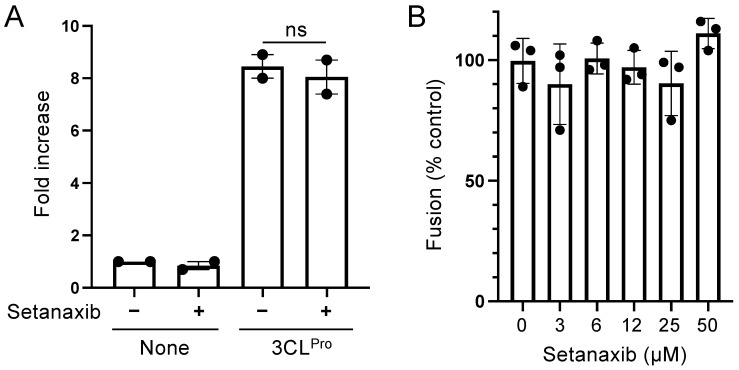
Setanaxib does not affect 3CL^Pro^ activity or viral fusion. (**A**) Huh7.5 cells expressing the 3CL^Pro^ luminescent biosensor were transfected with an expression plasmid that was either empty (None) or encoding SARS-CoV-2 3CL^Pro^ as described in Figure 2. After 5 h of incubation, Setanaxib was added at 25 μM. After 24 h of culture, luciferase activity in culture wells was determined and normalized to cell viability determined using the CellTiter-Glo reagent. Data correspond to means ± SEM from 2 independent experiments in triplicate and statistical significance was determined by Student’s *t*-test. (**B**) The potential impact of Setanaxib on membrane fusion induced the S protein of SARS-CoV-2 was determined in a cell-to-cell fusion assay. HEK-293T cells expressing both the S protein and the α peptide of β-galactosidase were co-incubated with HEK-293T cells expressing ACE2 and the Ω peptide of β-galactosidase. Cells were co-cultured for 5 h with increasing concentrations of Setanaxib to permit fusion, and β-galactosidase activity was determined using a bioluminescent substrate. Data correspond to means ± SEM from one experiment in triplicate and statistical significance was determined by Student’s *t*-test.

Setanaxib was developed as a NOX1/4 inhibitor but it appears to be effective on a SARS-CoV-2 infection assay for which relies on 3CL^Pro^ protease activity. Accordingly, this compound could also be an inhibitor of this viral enzyme. To check this hypothesis, Huh7.5 cells expressing the 3CL^Pro^ biosensor were transfected with a plasmid encoding SARS-CoV-2 3CL^Pro^ and cultured for 24 h with Setanaxib at 25 μM. 3CL^Pro^ expression activated the luciferase biosensor as expected, but similar levels of activation were obtained with or without Setanaxib (Figure 7A). This demonstrated that this compound does not inhibit the protease activity of 3CL^Pro^.

Setanaxib could also inhibit the interaction of the viral glycoprotein Spike with its receptor ACE2 or membrane fusion events [33]. This was evaluated in a membrane fusion assay recently adapted to this virus [24]. HEK-293T cells were transfected with plasmids to express either the Spike protein or its cellular receptor ACE2 together with complementary fragments of β-galactosidase. Cells were mixed to induce cell-to-cell fusion. Trans-complementation of β-galactosidase is associated to syncytia formation and can be used to quantify fusion events. β-galactosidase activity was not affected by the addition of Setanaxib in culture wells, thus demonstrating that Spike interaction with its receptor and membrane fusion events are not affected by this drug (Figure 7B). Because current SARS-CoV-2 variants are mainly characterized by variations in the glycoprotein, and thus on attachment to the ACE2 receptor and fusion capacity [34,35], these results suggest that they should also be susceptible to Setanaxib. Following these two control experiments, we can conclude that Setanaxib inhibits other step(s) of SARS-CoV-2 replication cycle and their identification will require further investigations.

## 4. Discussion

We have developed and validated an in vitro luminescent assay relying on the detection of 3CL^Pro^-mediated proteolysis as a reporter for SARS-CoV-2 infection. This assay was used to screen a metabolism-oriented chemical library to identify antiviral compounds which could potentially be transferred to clinic. In the top compounds found was a known SARS-CoV-2 inhibitor (Vidofludimus) [29] as well as molecules already suspected to impair the replication of this virus (Tretinoin; Clarithromycin) [4,32]. Most interestingly, we also identified Setanaxib, a well-characterized NOX1/4 inhibitor that was reported as a hit in a recently published chemical screening for SARS-CoV-2 inhibitors [6]. We thus further investigated and validated the antiviral properties of this molecule in different cellular models.

The urgent need for drugs inhibiting SARS-CoV-2 has motivated the development of screening assays to identify antivirals. Out of these, the simplest rely on in vitro measures of cytopathic effects induced by the virus [4,5]. Recombinant viruses expressing fluorescent proteins or luciferases as reporters were also developed [36,37]. High-throughput ELISA-based assays for detecting viral proteins in culture supernatants were also adapted to the screening of chemical libraries [38]. More focused system to identify drugs specifically targeting viral entry, components of the viral transcription/replication machinery or viral proteases PL^Pro^ and 3CL^Pro^ were also engineered [39,40,41,42,43,44,45,46,47,48]. In this work, we took advantage of luciferase-based assays previously described to monitor the proteolytic activity of PL^Pro^ and 3CL^Pro^ from MERS-CoV and PEDV [10,11]. As opposed to a recently described cellular assay in which the proteolytic cleavage of the reporter luciferase by SARS-CoV-2 3CL^Pro^ abolishes bioluminescence [44], the cleavage of our biosensor relaxed circularly-permuted domains of luciferase to actually trigger bioluminescence. The same principle was recently applied by others to EGFP or luciferase for detecting the proteolytic activity of 3CL^Pro^ from SARS-CoV-2 [12,45]. However, and compared to previous reports, we achieved a demonstration that our assay can detect 3CL^Pro^ activity in the context of SARS-CoV-2 infection, thus allowing the selection of drugs inhibiting not only 3CL^Pro^ but different steps of the virus replication cycle. The same assay could be used to evaluate the antiviral activity of neutralizing antibodies and biologics against SARS-CoV-2 [49].

We took advantage of this assay to screen an original library of metabolic modulators for an antiviral effect. Indeed, it is now well-established that coronaviruses interact with several cellular metabolic enzymes and reprogram a large array of metabolic pathways to promote their replication [50,51]. Consequently, these pathways represent promising targets for developing host-directed antivirals [52]. In the list of hit compounds, Vidofludimus can be viewed as a proof of concept for our assay and screening strategy. Indeed, Vidofludimus is an inhibitor of dihydroorotate dehydrogenase (DHODH), the fourth and rate-limiting step in the de novo pyrimidine biosynthesis pathway. Inhibitors of this metabolic pathway deprive cells in pyrimidines and block their proliferation. For this reason, DHODH inhibitors have been developed for treating cancer and autoimmune diseases [53]. Most interestingly, these drugs also exhibit a broad-spectrum antiviral activity in vitro that is now well documented [54]. However in vivo, divergent results were obtained in virus-infected animal models. Nevertheless, this dual antiviral and immunomodulatory activity of DHODH inhibitors suggest some potential benefit in the treatment of Covid19 [55]. We have previously reported that HCoV-229E, a member of the alphacoronavirus genus, is sensitive to DHODH inhibition [56]. Furthermore, several recent reports have shown that SARS-CoV-2 replication is impaired in cells treated with DHODH inhibitors such as teriflunomide, Vidofludimus or PTC299 [29,57,58]. Several DHODH inhibitors including Vidofludimus are clinically evaluated in the treatment of Covid19 (ClinicalTrials.gov Identifier: NCT04361214; NCT04379271; NCT04425252; NCT04575038; NCT04439071; accessed on 22 July 2021).

Our results confirm the inhibitory effect of Vidofludimus on SARS-CoV-2 replication, and we extended this observation to BAY2402234, a potent DHODH inhibitor currently developed by Bayer as a treatment of acute myeloid leukemia [31]. Clinical trials started recently to evaluate the benefit of DHODH inhibitors in the treatment of SARS-CoV-2. These should provide important conclusions on the therapeutic potential of these drugs against coronavirus infections. Clarithromycin was also in the short list of SARS-CoV-2 inhibitors identified in the screen. Clarithromycin is a macrolide antibiotic closely related to Azithromycin, a drug previously reported for blocking SARS-CoV-2 replication in vitro [4,59]. However, a large fraction of SARS-CoV-2 inhibitors identified by functional screening such as hydroxychloroquine and macrolide antibiotics are cationic-amphiphilic molecules which will accumulate in endosomes and lysosomes (and are therefore referred as “lysosomotropic”). Once in the lysosomes, these drugs are trapped by protonation and this leads to an increased pH, a perturbed endolysosomal trafficking, and an induction of phospholipidosis [60,61,62,63]. As a consequence, not only the pH-dependent entry of SARS-CoV-2 through the endolysosomal pathway but also later steps of the viral replication cycle which depend on intracellular vesicles are impaired [59,62]. Even if conflicting results have been published on the efficacy of these drugs in patients, when assessed in macaques in a highly controlled experiment setting, azithromycin combined with hydroxychloroquine did not show any protection against infection with SARS-CoV-2 [64]. 

Our screen also identified Tretinoin, the acid form of vitamin A and a RAR agonist, as an inhibitor of SARS-CoV-2 replication in line with Riva et al. [5]. This molecule was suggested to block the ion channel formed by SARS-CoV-2 envelope (E) protein [32]. However, this may not be the actual mode of action of tretinoin since the inhibition of SARS-CoV-2 replication by Tazarotene, another RAR agonist, is reversed by the RAR antagonist Ro41-5253 [5]. This suggests that antiviral properties of RAR agonists such as Tretinoin rely on RAR transcriptional activity and the modulation of target genes. RIG-I (retinoic-acid induced gene I; DDX58) which is a key sensor for viruses and an essential component of the host antiviral response is upregulated by retinoids. However, the Huh7.5 cell line that was used for the screen is mutated for this antiviral factor. It has also been reported that SARS-CoV-2 receptor ACE-2 is downregulated by retinoids [65,66]. Accordingly, the suppression of ACE-2 could contribute to the inhibition of SARS-CoV-2 replication by RAR agonists. Ongoing clinical trials are evaluating the benefit of RAR agonists in the treatment of Covid19 (ClinicalTrials.gov Identifier: NCT04361422; accessed on 22 July 2021).

Finally, we report the inhibition of SARS-CoV-2 by Setanaxib. This molecule is a NOX1/4 inhibitor developed to prevent oxidative stress and fibrosis in multiple diseases including primary idiopathic fibrosis, primary biliary cholangitis, and diabetic nephropathy [67]. Because Setanaxib is highly active against NOX1/4 and is a well-tolerated drug exhibiting a good pharmacokinetic, it has been extensively evaluated in pre-clinical in vivo models and several clinical trials have been performed or are ongoing. Biering SB et al. have recently reported the inhibition of SARS-CoV-2 by Setanaxib in Calu3 and HPMEC cells transduced for ACE2, but this molecule was found inactive in Huh7 and Vero E6 cells [6]. Here, we showed that Setanaxib is active in Huh7.5 cells, Beas-2B cells transduced for ACE2 and most importantly, human primary nasal epithelial cells. Therefore, the antiviral effect of Setanaxib appears to be cell-type specific. Surprisingly, opposite results were obtained in Huh7 and the related clone Huh7.5, but it should be emphasized that these two cell lines do differ in their susceptibility to SARS-CoV-2 infection [8]. The difference between these two cell lines in regards to SARS-CoV-2 infection is likely to extend beyond the well-characterized mutation in RIG-I.

The mechanism responsible for the inhibition of SARS-CoV-2 infection by Setanaxib remains elusive. NOX proteins are transmembrane enzymes that generate reactive oxygen species (ROSs) in different subcellular compartments including cellular membranes, lysosomes, mitochondria and the endoplasmic reticulum [67]. In physiological conditions, ROS generated by NOX enzymes participate to an antimicrobial response but also to cell signaling and post-translational modifications of proteins. Interestingly, ROS production is increased in monocytes infected by SARS-CoV-2 and this burst is essential to viral replication [68]. Although complex I of the respiratory chain was identified as the main source of ROS in this system, it is possible that NOX enzymes support SARS-CoV-2 replication by producing ROS in other cellular systems. The NOX enzymes are also known for regulating the pH of lysosomes. Indeed, elevated oxidative activity has been linked to lysosomal alkalization [69]. To determine the effect of Setanaxib on the lysosomal compartment, we performed the following exploratory experiment. Vero cells were treated with Setanaxib and grown for 4 h and then the lysosomes were stained with LysoTracker Deep Red. When assessing hydroxychloroquine, which was used as a reference lysosomotropic drug associated to phospholipidosis [63], a massive accumulation of large intracellular vesicles that stained positive with LysoTracker Deep Red was observed (Supplementary Figure S3). On the other hand, Setanaxib did not induce a similar phenotype but increased the intracellular staining with the lysosomal dye. This suggests that Setanaxib also interferes with the lysosomal compartment and this could account for an antiviral activity [62]. Deciphering the mechanisms involved and the role of NOX enzymes requires further investigations. 

In conclusion, we report here a functional cell-based assay to identify inhibitors of SARS-CoV-2 infection which can be adapted to all strains of SARS-CoV-2 and potentially other coronaviruses without the need of introducing reporter genes in the viral genome. The screening of a metabolism-oriented library led to the identification of several hits including the DHODH inhibitor Vidofludimus and the NOX1/4 inhibitor Setanaxib which antiviral activity was validated in PNECs. Although further investigations are required to determine the mechanisms involved in this antiviral activity, our data suggest that this class of molecule may have potential against SARS-CoV-2 infection. Interestingly, it has been shown that NOX activation is associated with inflammation and severe Covid19, and the potential of NOX inhibitors for controlling the cytokine storm induced by SARS-CoV-2 has also been discussed [70]. Therefore, Setanaxib could show a dual benefit in patients by limiting inflammation and by blocking viral replication as presented in this report.

## Figures and Tables

**Table 1:** Primers used for RT-qPCR.

Primer	Primer (5′–3′)
SARS-CoV-2 N for	AAACATTCCCACCAACAG
SARS-CoV-2 N rev	CACTGCTCATGGATTGTT

**Table 2:** Primers and probe used for RT qPCR.

Primer/Probe Name	Primer/Probe Sequence (5′–3′)
NCoV_AN_F	GGCCGCAAATTGCACAAT
NCoV_AN_R	CCAATGCGCGACATTCC
NCoV_AN_P	***FAM***-CCCCCAGCGCTTCAGCGTTCT-***BHQ1***

Reporter dye (FAM) and quencher (BHQ1) elements are indicated in bold and italics.

## Data Availability

The data presented in this study are available in the main text and the Supplementary Materials of this article.

## Supplementary Materials

The following are available online at https://www.mdpi.com/article/10.3390/v13091814/s1, Figure S1: Structures of the compounds used in this study. Figure S2: Inhibition of SARS-CoV-2 replication by DHODH inhibitors, Figure S3: Lysosomal staining in Setanaxib-treated cells. Table S1: Inhibitory effect of the 276 compounds showing no sign of cellular toxicity.
