# Supplementary material for: A Bioluminescent 3CLPro Activity Assay to Monitor SARS-CoV-2 Replication and Identify Inhibitors
Source: Viruses. 2021 Sep 12;13(9):1814. doi: 10.3390/v13091814 (PMC8473059; doi:10.3390/v13091814)
Supplement: Supplementary file 1 [file viruses-13-01814-s001.zip › viruses-1332330-supplementary.pdf]

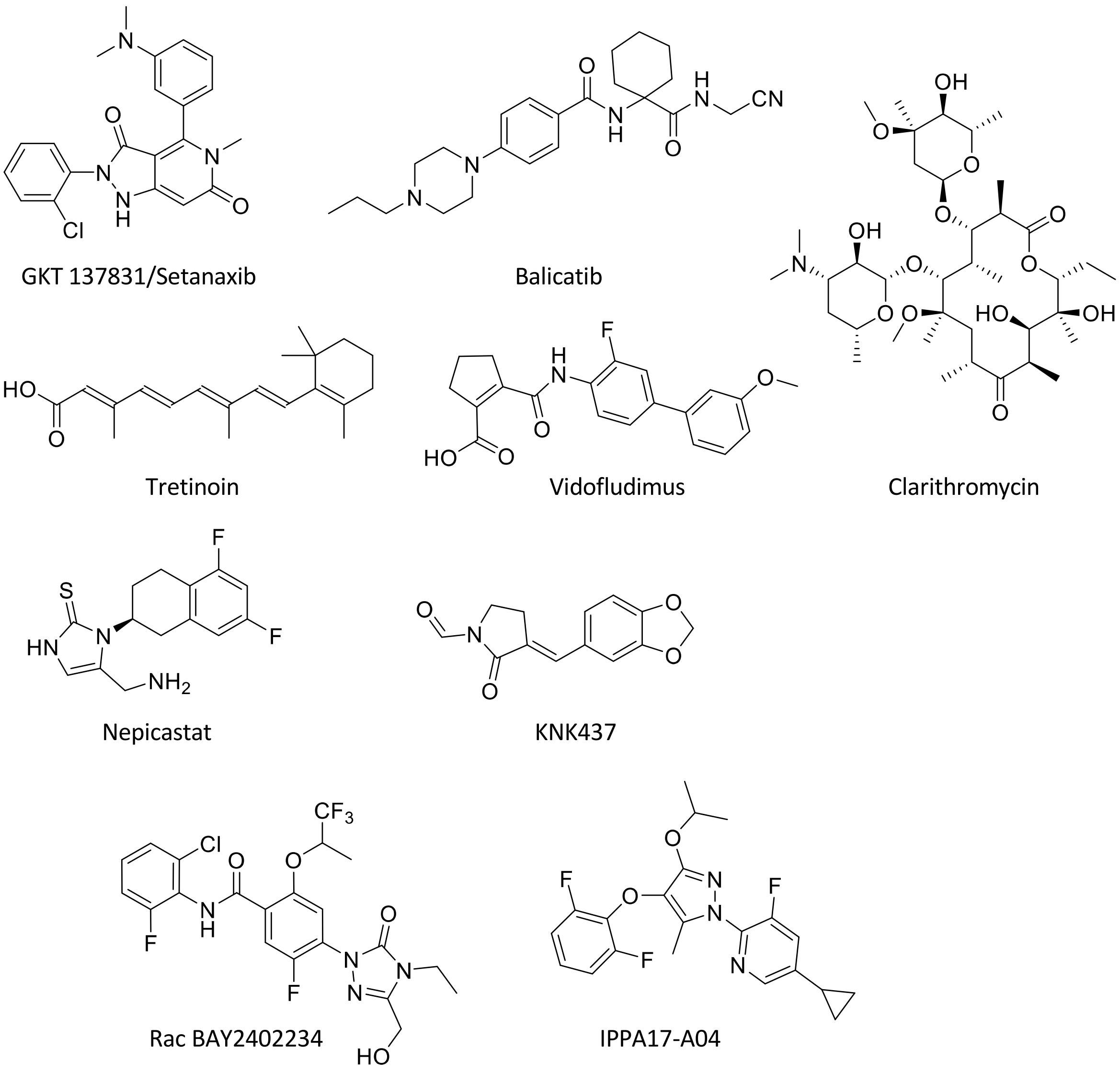

Figure S1. Structures of the compounds used in this study.

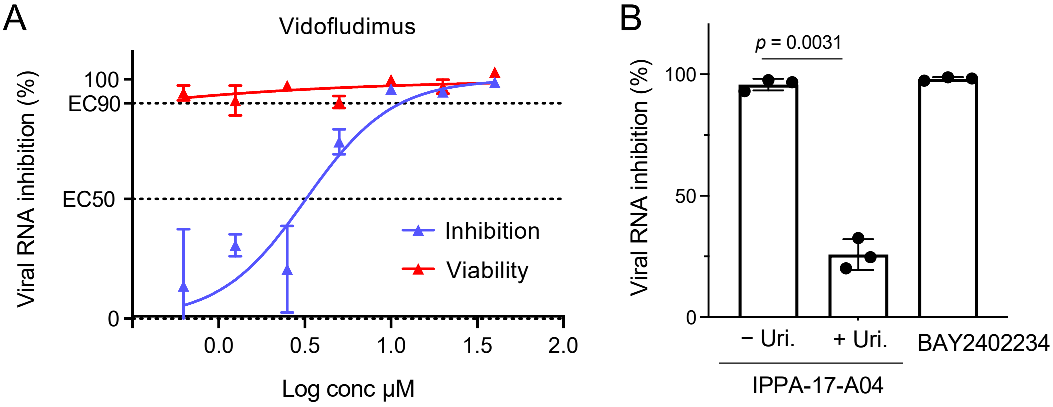

Figure S2. Inhibition of SARS-CoV-2 replication by DHODH inhibitors. (**A**) Vero cells were infected SARS-CoV-2 (MOI = 0.002) and cultured with increasing concentration of Vidofludimus or DMSO alone. After 48 h, culture supernatants were collected and viral particles quantified by RT-qPCR. The impact of Vidofludimus on cellular viability and proliferation was determined in uninfected cultures using the CellTiter-Blue reagent. Results were expressed as a percentage of inhibition relative to

control wells. (**B**) Vero cells were infected SARS-CoV-2 (MOI = 0.002) and cultured with DMSO alone, IPPA17-A04 (0.5 µM) in the absence or presence of uridine (120 µM) or BAY2402234 (0.6 µM). After 48 h, culture supernatants were collected and viral particles quantified by RT-qPCR. Data correspond to means ± SEM from one experiment in triplicate and statistical significance was determined by Student's t-test.

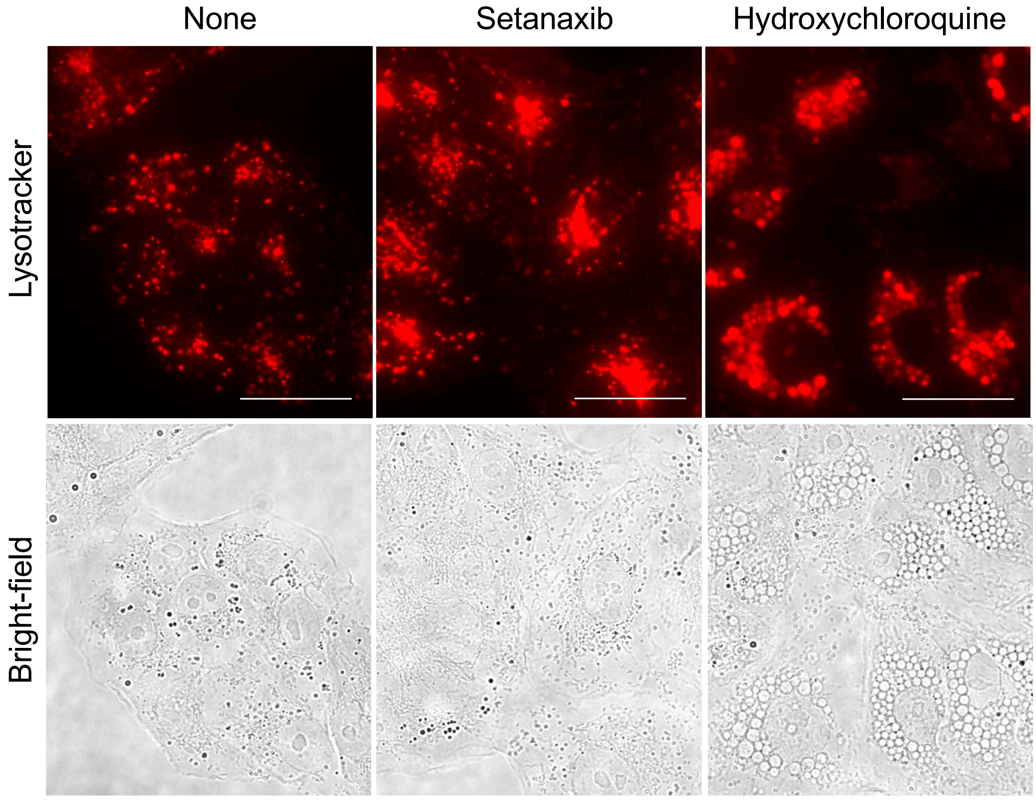

Figure S3. Lysosomal staining in Setanaxib-treated cells. To determine the potential impact of Setanaxib on lysosomes, Vero cells were cultured alone or with Setanaxib (25 µM). Hydroxychloroquine was used as a reference lysosomotropic drug (50 µM). After 4 h at 37 °C, lysosomes were stained with LysoTracker Deep Red and imaged by fluorescence microscopy. Scale bar is 20 µm.
